# Upgraded *LauePt4* for rapid recognition and fitting of Laue patterns from crystals with unknown orientations

**DOI:** 10.1107/S1600576723007926

**Published:** 2023-09-27

**Authors:** Vincent W. Huang, Yafei Liu, Balaji Raghothamachar, Michael Dudley

**Affiliations:** a Department of Materials Science and Chemical Engineering, Stony Brook University, Stony Brook, New York 11790, USA; HPSTAR and Harbin Institute of Technology, People’s Republic of China

**Keywords:** Laue diffraction, *LauePt4*, X-ray crystallography, indexing

## Abstract

The *LauePt* program is a popular and easy-to-use crystallography tool for indexing and simulating X-ray Laue patterns, but its previous versions lack search functions for recognizing Laue patterns taken from crystals with unknown orientations. To overcome this obstacle, a major upgrade of the program, called *LauePt4*, is presented with three robust search schemes implemented: (i) crystal rotation along a single diffraction vector, (ii) a look-up method to search for reflection pairs matching the interplanar angle of two selected diffraction spots, and (iii) a more efficient look-up scheme to search for reflection triplets matching three interplanar angles. Extensive tests show that all these schemes, together with the convenient graphical user interfaces and highly optimized computing algorithms, are reliable and powerful for recognizing and fitting Laue patterns of any crystal taken under any diffraction geometry.

## Abstract 2

An upgrade is presented to the popular *LauePt* program for indexing and simulating X-ray Laue patterns. The upgraded program allows for recognizing and fitting Laue patterns of any crystal type recorded under any diffraction geometry.

## 1. Introduction

The *LauePt* computer program is an interactive Windows application for simulating white-beam X-ray Laue patterns of general crystals. Since publication of its Version 3 (Huang, 2010 ▸), *LauePt* has become a very popular crystallography tool used by many researchers worldwide, particularly for synchrotron white-beam topography or diffraction, determination of crystal orientations by the Laue method, and crystallography education (Oriwol *et al*, 2013 ▸; Woodworth *et al.*, 2014 ▸; Asadchikov *et al.*, 2018 ▸; Danilewsky, 2020 ▸; Cui *et al.*, 2020 ▸; Zolotov *et al.*, 2020 ▸). As a small program with all the functions integrated into a single executable file, *LauePt* has a complete set of convenient and straightforward graphical user interfaces (GUIs) with which one can quickly simulate a complicated Laue pattern with just a few mouse clicks. Therefore, it can be used even by non-specialists with little crystallography knowledge or training.

Laue patterns generated by *LauePt* can be directly compared with real patterns (Laue images) recorded by X-ray imaging detectors or X-ray films. *LauePt* does not have a procedure to process raw Laue images into maps of diffraction spot positions. Instead, it allows the user simply to import a recorded Laue image as the background, and the simulated pattern is plotted directly on top of it for interactive visual comparison. The program can handle any ordinary images in TIFF (8-bit), JPEG and BITMAP formats. This feature dramatically reduces the complexity of Laue pattern simulation and fitting compared with the large-scale synchrotron Laue microdiffraction programs (Barabash *et al.*, 2001 ▸; Liu *et al.*, 2004 ▸; Gupta & Agnew, 2009 ▸; Ulrich *et al.*, 2011 ▸; Robach & Micha, 2015 ▸; Zhang *et al.*, 2017 ▸; Örs *et al.*, 2018 ▸; Morawiec, 2020 ▸; Purushottam Raj Purohit *et al.*, 2022 ▸; Gürsoy *et al.*, 2022 ▸; Seret *et al.*, 2022 ▸;). *LauePt* has another unique advantage, namely accurate intensity calculations (based on the kinematic diffraction model). Consequently, the visual comparison of both diffraction spot positions and intensities is clear and vivid, and the pattern fitting, once achieved, has no ambiguity even for poor-quality recorded Laue images. In addition to the *hkl* Miller indices, each spot of the simulated pattern also gives information about the wavelengths, structure factors and intensities of all the harmonics based on the incident spectrum. The fast and accurate intensity calculations of *LauePt* may potentially enable simple and rapid white-beam diffraction techniques to have new capabilities for verifying or refining crystal structures, or even solving crystal structures.

Unfortunately, however, the previous versions of *LauePt* have a severe shortcoming in a lack of searching/fitting procedures. To fit a recorded image, the user must make initial guesses of two perpendicular directions of the crystal in laboratory space. Starting from these directions, *LauePt* can freely rotate the crystal around three orthogonal axes and the simulated pattern changes instantly with rotation. For crystals with a roughly known orientation, one can quickly find a simulation matching the real image by these rotation operations, from which the exact 3D crystal orientations and the surface miscut can be derived (Huang, 2010 ▸). Unfortunately, for crystals with unknown orientations, it becomes extremely difficult, if not impossible, for *LauePt* to find from millions of possible patterns a correct one that matches the recorded Laue image. This has been a major obstacle to its many applications.

In this paper, we introduce a major upgrade of *LauePt* to Version 4 by implementing three fast and reliable search schemes to overcome this obstacle. These improvements make *LauePt4* a full-function program without affecting its compactness and ease of use.

**Figure 1:**
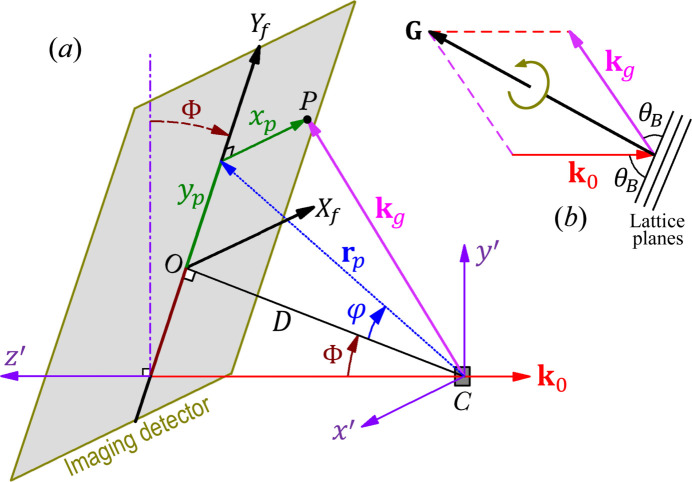
(*a*) A schematic diagram of Laue diffraction geometry. *C* is the crystal. *O* is the perpendicular foot of *C* on the imaging detector plane. *D* is the imaging distance from *C* to *O*. The *X*_*f*_ axis is along the opposite direction to *x*′. *LauePt* allows the detector to be rotated around *x*′ by an angle Φ ( 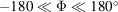 ). [Φ = 90° corresponds to the reflection geometry widely used in Laue microdiffraction where the X-ray imaging detector is placed above the crystal (Gürsoy *et al.*, 2022 ▸; Ulrich *et al.*, 2011 ▸), while Φ = 180° corresponds to the forward transmission geometry.] (*b*) Crystal rotation along the diffraction vector **G** without affecting the diffracted wavevector **k**_*g*_.

The Laue images in this paper were recorded with a laboratory Laue camera in the back-reflection geometry [Φ = 0 in Fig. 1 ▸(*a*)]. The source is an AEG Cu X-ray tube (model FK 60-10) operated at 30 kV and 30 mA, and the incident beam is limited by a 1 mm diameter pinhole. The field of view of the imaging detector (a 2009 Photonic Science X-ray backscattering dual CCD detector) is 145 mm by 96 mm and the pixel size is ∼70 µm. The Laue images presented here are 8-bit grayscale TIFF images (with 256 intensity levels) recorded with a typical exposure time of around 3 min. State-of-the-art Laue cameras may have much shorter exposure times and better image contrast. Currently *LauePt4* only supports 8-bit color depth, which is adequate for computer screen display and visual comparison.

## 2. Crystal rotation along a specific diffraction vector

As shown in Fig. 1 ▸(*a*), *LauePt* sets up a fixed *x*′*y*′*z*′ laboratory coordinate system (CS), where the *z*′ axis is opposite to the incident direction and the *x*′ axis is along the horizontal direction. Crystal rotation is performed around any of the three axes *X*, *Y* and *Z* that initially coincide with *x*′, *y*′, *z*′, respectively. Here a brute-force algorithm for searching a Laue pattern is a triple-nested loop of rotation around *X*, *Y*, *Z* with a step size of 2°, but that would require (180 × 180 × 180) ≃ 6 000 000 simulations and visual comparisons, which is impractical.

**Figure 2:**
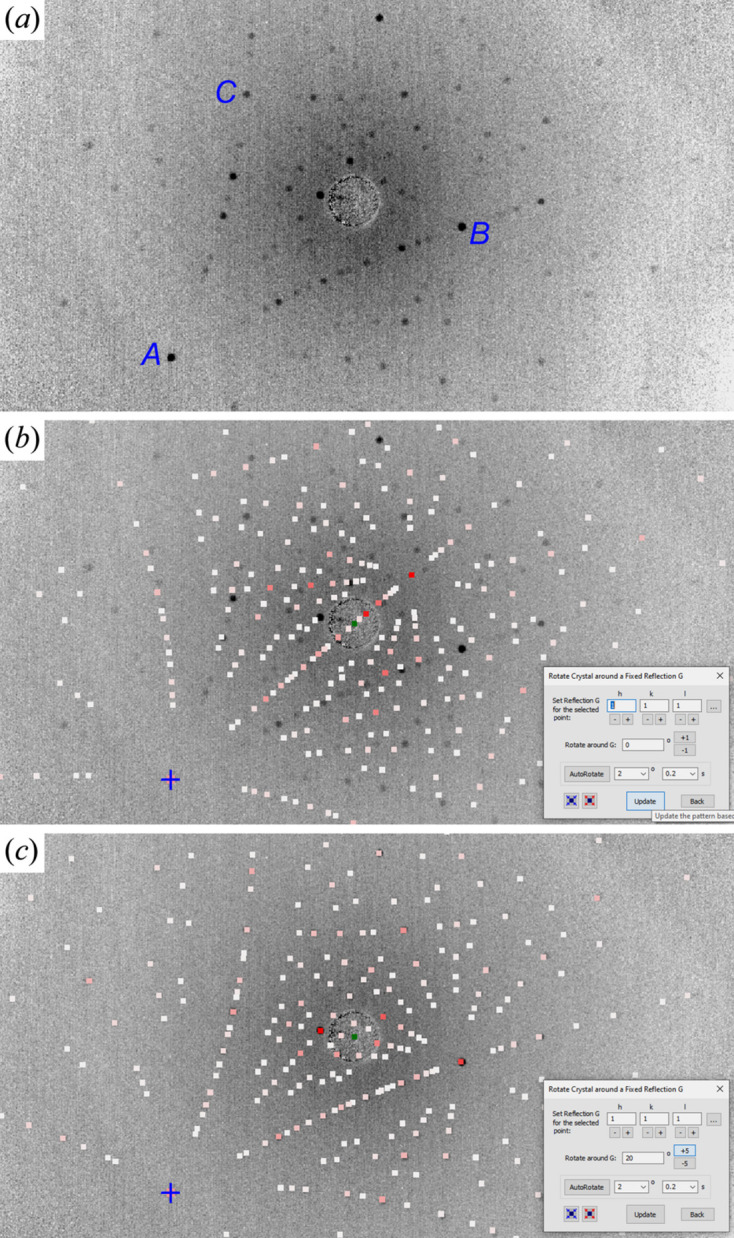
(*a*) Back-reflection Laue image taken from an Si wafer. (*b*) Laue pattern simulated by assigning the **G** = 111 reflection to spot *A*. (*c*) A matching pattern simulated with 20° crystal rotation around **G** from panel (*b*), which determines the wafer surface to be (431). Image width 141 mm and imaging distance *D* = 36 mm. The white to red spot colors indicate weak to strong diffraction intensities.

A common feature of Laue diffraction is that a low-index reflection (such as 100, 110, 111 *etc.*) usually appears as an isolated diffraction spot, which is also the intersection of different hyperbolas or ellipses formed by diffraction spots of different zones. For example, Fig. 2 ▸(*a*) is a back-reflection Laue image of an Si wafer with an initially unknown orientation, where *A*, *B* and *C* are such isolated spots. On the basis of this phenomenon, our first strategy is that, if a Laue image contains a well isolated spot, we assign it a low index as a trial. (If there are no obvious isolated spots in the image, one can easily rotate the crystal during the experiment to bring one or more isolated spots onto the imaging detector.) Next, we design a scheme, called the G-rotation method, to rotate the crystal around the diffraction vector **G** of this spot. As indicated in Fig. 1 ▸(*b*), crystal rotation around **G** does not change the specific diffracted wavevector **k**_*g*_ (= **k**_0_ + **G**) since both **G** and the incident wavevector **k**_0_ remain unchanged during rotation. Consequently, the position of the selected spot *P* also remains fixed on the detector plane in Fig. 1 ▸(*a*) during rotation. However, other spots will (dramatically) change positions during rotation.

*LauePt4* allows the user to right click on a diffraction spot *P* to select it. On the basis of the mouse click position on the screen, the program calculates the real position (*x*_*p*_, *y*_*p*_) of *P* in the Cartesian CS *X*_*f*_ *Y*_*f*_ of the detector plane. From the geometry in Fig. 1 ▸(*a*) the diffracted wavevector is 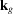 = 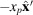 + 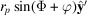 + 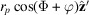 in the *x*′*y*′*z*′ laboratory CS, where 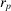 = 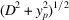 , φ = 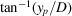 and 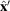 , 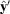 , 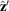 are unit vectors along the *x*′, *y*′, *z*′ axes, respectively. Here only the direction of **k**_*g*_ is valid, represented by the unit vector 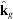 = 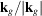 . The unit vector along the incident direction is 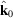 = 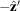 . The **G** vector in Fig. 1 ▸(*b*) is parallel to the vector 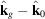 . *LauePt4* then obtains a unit vector 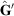 = 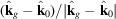 along **G** in the *x*′*y*′*z*′ laboratory CS. The program also defines another unit vector 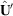 = 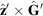 perpendicular to 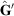 .

After the selection, the user can assign a Miller index *hkl* to *P*, *i.e.* the diffraction vector of *P* is **G** = *h* a* + *k* b* + *l* c* in the *crystal* CS, where a*, b*, c* are the three primitive reciprocal-lattice vectors in the crystal CS. The program also automatically selects another diffraction vector **G**_*u*_ = *h*_*u*_ a* + *k*_*u*_ b* + *l*_*u*_ c* parallel to 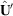 . Afterwards, the two directions **G** and **G**_*u*_ completely determine the crystal orientation in the *laboratory* CS (see the supporting information for the transformation between the crystal and laboratory CSs). *LauePt4* simulates the pattern based on (*hkl*) and (*h*_*u*_ *k*_*u*_ *l*_*u*_) in the laboratory CS. Then **G**_*u*_ can be rotated around **G** by 360°, which corresponds to a crystal rotation around **G**. Therefore, if the guessed index *hkl* is correct, one can find the matching simulation within the 360° rotation. If the search fails, one can try another low index to repeat this process.

For example, the simulation of Fig. 2 ▸(*b*) is generated by right clicking spot *A* in Fig. 2 ▸(*a*) and assigning it the Miller index 111. Here the simulation does not match the recorded image, but the calculated 111 reflection position marked by the ‘+’ symbol indeed coincides with spot *A*. When the crystal is rotated along **G** = 111, the simulated pattern changes, but the 111 reflection spot ‘+’ is fixed at spot *A*, as expected. After rotation of 20°, the simulated pattern successfully matches the recorded pattern in Fig. 2 ▸(*c*). Under this condition, *LauePt4* gives the ‘absolute orientation’ of the crystal plane perpendicular to *z*′ as (1, 0.739, 0.254). In other words, the *z*′ direction is along vector a* + 0.739b* + 0.254c* in reciprocal space. (We allow the indices to be fractions in order to express arbitrary orientations that are not necessarily along discrete reciprocal-lattice vectors.) The plane (1, 0.739, 0.254) is 0.46° away from the true lattice plane (431). Since the Si wafer was perpendicular to *z*′ in the experiment, the wafer surface is determined to be very close to (431) only with a small miscut angle of 0.46°. *LauePt4* also gives the orientations of the crystal along the *x*′ and *y*′ directions. Here spot *B* is determined to be reflection 011 (actually its harmonics) in Fig. 2 ▸(*c*). One may alternatively select this spot and assign 011 to it. Then rotating the crystal around the corresponding vector **G** = 011 can also easily reach the same matching pattern.

Our extensive tests show that the simple G-rotation method is surprisingly successful even for complicated patterns with high-density spots, although it may take a number of attempts to try the index of the selected spot before the fitting is achieved. It works particularly well for separating different sets of Laue patterns (diffracted from crystal twinning, domains or grains) superimposed on a single Laue image, since this method only needs a single spot to simulate an entire Laue pattern. In contrast, in the following methods one can possibly select two or three spots belonging to different Laue patterns in the superimposed image.

## 3. Looking up lattice plane pairs with a selected interplanar angle

The second fitting procedure added to *LauePt4* is the classical method of using a look-up table (LUT) method to search for pairs of lattice planes (reflections) satisfying the interplanar angle of two selected diffraction spots (Heizmann & Baro, 1967 ▸; Riquet & Bonnet, 1979 ▸). Here we call it the LUT-P method. Implementation of LUT-P in *LauePt4* is very simple. First, the user selects two spots (low-index isolated spots are always preferable, if possible) in the imported Laue image. *LauePt4* immediately calculates the two diffraction vectors **G**_1_ and **G**_2_ in the laboratory CS using the method in Section 2, from which the interplanar angle 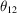 = 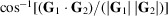 is obtained. *LauePt4* actually does not have an LUT calculated in advance. Instead, it instantly calculates the angle θ between each possible reflection pair **H**_1_ = *h*_1_ a* + *k*_1_ b* + *l*_1_ c* and **H**_2_ = *h*_2_ a* + *k*_2_ b* + *l*_2_ c* (in the crystal CS) within the index range limited by |*h*_1,2_| ≤ *H*_max_, |*k*_1,2_| ≤ *K*_max_, |*l*_1,2_| ≤ *L*_max_. If |θ − θ_12_| ≤ Δθ, *LauePt4* adds the pair (*h*_1_ *k*_1_ *l*_1_, *h*_2_ *k*_2_ *l*_2_) to the candidate list. Here Δθ is the angular tolerance set by the user.

After all the candidate pairs have been found, *LauePt4* allows the user to select any pair (**H**_1_ = *h*_1_ *k*_1_ *l*_1_, **H**_2_ = *h*_2_ *k*_2_ *l*_2_) from the list to simulate the corresponding pattern. Since assigning **H**_1_ and **H**_2_ to the selected **G**_1_ and **G**_2_ vectors completely decides the crystal orientation in the laboratory CS, the simulation is unique for each pair, but it will not necessarily match the recorded image, except that the simulated **H**_1_ and **H**_2_ reflection spots will always coincide with the two selected spots. Therefore, the user must find the correct pair (**H**_1_, **H**_2_) with which the entire simulation matches the recorded image. Here **H**_1_ and **H**_2_ must also be in the correct order, *i.e.* swapping the reflection indices between the two spots will give a different pattern. The simulation process is similar to that used in Section 2, where one only needs to replace **G** and **G**_*u*_ in Section 2 with **H**_1_ and **H**_1_ × **H**_2_, respectively (see the supporting information).

**Figure 3:**
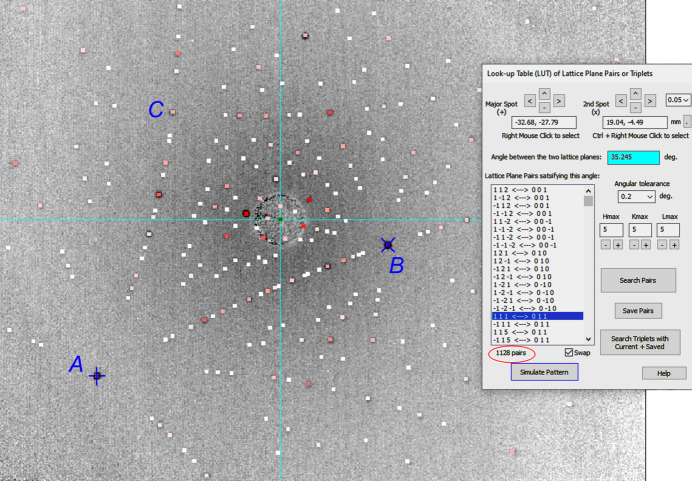
LUT-P search performed on the diffraction spots *A* and *B* gives the correct simulation matching the recorded image [the same as Fig. 2 ▸(*a*)].

Fig. 3 ▸ shows the Laue pattern simulated by selecting spots *A* and *B* in Fig. 2 ▸(*a*). Here the two spots are selected by simple right mouse clicks on the computer screen. *LauePt4* then calculates the angle between the two corresponding reflections as θ_12_ = 35.245°. Here, with *H*_max_ = *K*_max_ = *L*_max_ = 5 and Δθ = 0.2°, *LauePt4* finds 1128 reflection pairs satisfying this interplanar angle, which is a large collection (reducing the tolerance to Δθ = 0.1° can reduce the number of pairs to 768). The indices of *A* and *B* have been identified as 111 and 011, respectively, in Fig. 2 ▸, and the pair (111, 011) is indeed included in the list as a candidate. Afterwards, the user can try any pair in the list to let *LauePt4* simulate the corresponding pattern. The simulation in Fig. 3 ▸ is based on the pair (111, 011), *i.e.* *A* and *B* are assigned indices 111 and 011, respectively. The simulation almost perfectly matches the Laue image and all the results are consistent with those of Fig. 2 ▸(*c*).

Note that all the symmetry-related equivalent pairs are included in the list of Fig. 3 ▸ [*e.g.* (111, 011), (111, 110), (111, 101), ( 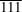 , 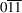 ),…]. If the user selects any pair other than these equivalents, the simulation will not match the Laue image. For the current LUT-P method, therefore, the user needs to test the candidates in the list one by one (including the swapped pairs). The user can use the mouse to click any pair in the list to select it (see the highlighted pair in the list of Fig. 3 ▸), and then *LauePt4* immediately simulates the Laue pattern based on the selected pair and updates the computer screen with the current simulation on top of the recorded image. In addition to selection by mouse click, *LauePt4* also allows the user to use the UP (↑) and DOWN (↓) arrow keys of the computer keyboard to change the selection in the list. In other words, the user can keep pressing the UP or DOWN arrow key to let the selection move up or down quickly over each item in the list. During this scrolling process, *LauePt4* instantly makes the corresponding simulation and screen update based on the transient selection. Since it computes the patterns extremely quickly due to the highly optimized algorithms and code, *LauePt4* is always capable of providing an instant screen update of the changing simulation even if the scrolling is very fast. This feature greatly reduces the time to search for the correct pair when the list is very long. Therefore, LUT-P runs fast in *LauePt4*. More importantly, this method is reliable and seldom fails.

## 4. Advanced look-up method for matching triplets

To improve the LUT-P efficiency further, we have developed an advanced look-up technique, called the LUT-T method, to search for *reflection triplets* satisfying three interplanar angles. The principles are again very simple. As an example, let us first select spots *A* and *C* in Fig. 2 ▸(*a*) to perform the LUT-P search described in Section 3. *LauePt4* gives a list of 1392 pairs (not including the swapped pairs). *LauePt4* can save this list of pairs as 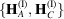 and the interplanar angle θ_*AC*_ between *A* and *C*. Next, we keep *A* selected (as the major spot) and select *B* as the second spot (with *C* deselected) to perform LUT-P again. As has been shown in Fig. 3 ▸, this step gives 1128 potential pairs, denoted 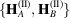 . The interplanar angle between *A* and *B* is θ_*AB*_. *LauePt4* also calculates the interplanar angle between *B* and *C*, θ_*BC*_.

**Figure 4:**
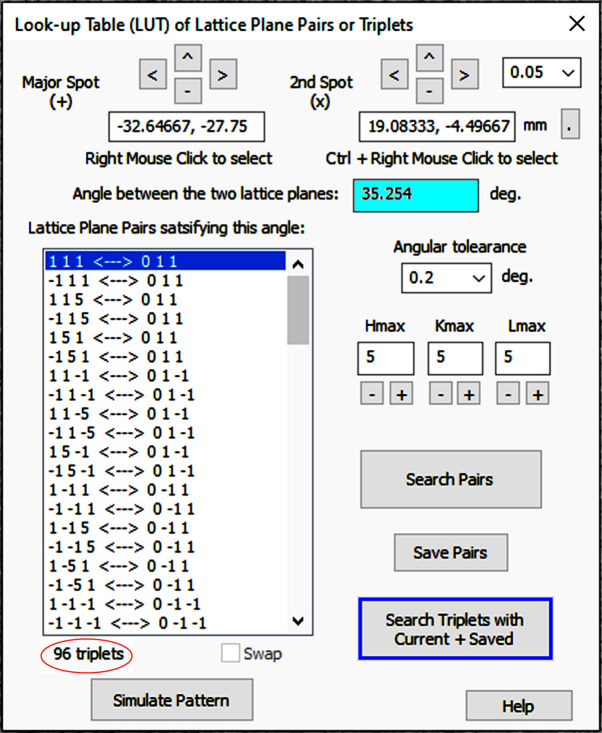
The dialog for LUT-P and LUT-T searches. The list box has the 96 triplets obtained from the LUT-T search performed on spots *A*, *B* and *C* in Fig. 2 ▸(*a*).

In the LUT-T procedure, *LauePt4* compares each reflection pair in 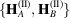 with all the saved pairs in 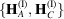 . During the comparison, if two pairs 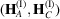 and 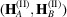 have a common reflection, *LauePt4* calculates the angle θ_23_ between the other two reflections [for example, between 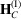 and 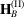 if 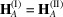 ]. If θ_23_ is the same as θ_*BC*_ within the angular tolerance, the three different reflections form a triplet satisfying the three interplanar angles θ_*AC*_, θ_*AB*_ and θ_*BC*_. Then the pair 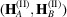 is kept in 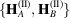 . If θ_23_ is different from θ_*BC*_, the pair is discarded. *LauePt4* also discards the pair 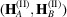 if all the pairs in 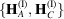 do not have a common reflection with this pair. As shown in Fig. 4 ▸, after this filtering process, the number of pairs in 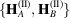 is dramatically reduced from 1128 to 96. Further examination shows that the list in Fig. 4 ▸ only contains three different configurations: ( 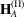 = 111, 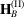 = 011), (115, 011) and (115, 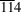 ). All the other pairs are symmetry-related equivalents to these three pairs. Now we can let *LauePt4* simulate the patterns of these three pairs, from which the (115, 011) and (115, 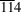 ) pairs are readily excluded since the corresponding patterns do not match the recorded Laue image. The remaining pair (111, 011) is the correct solution, which is consistent with Figs. 2 ▸ and 3 ▸.

**Figure 5:**
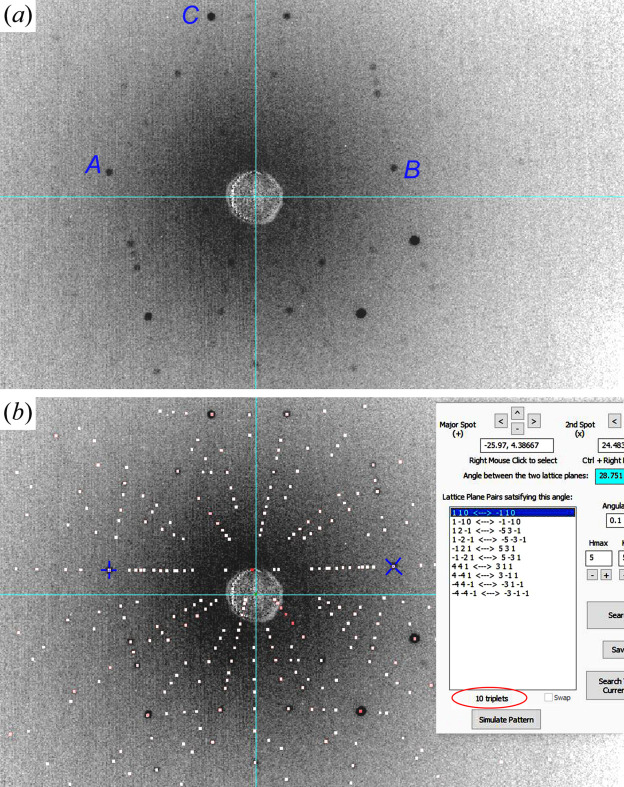
The LUT-T search and pattern simulation of a β-Ga_2_O_3_ crystal. (*a*) Recorded back-reflection Laue image of the crystal. (*b*) Ten reflection triplets obtained from the LUT-T search for spots *A*, *B*, *C* in panel (*a*) and the matching simulation based on the valid solution (110, 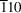 ). Imaging distance *D* = 45.8 mm, *H*_max_ = *K*_max_ = *L*_max_ = 5 and Δθ = 0.1°. Image width 112 mm.

As another example, Fig. 5 ▸ shows the application of LUT-T to a β-Ga_2_O_3_ crystal with much lower symmetry than Si. β-Ga_2_O_3_ has a monoclinic structure with unit-cell parameters *a* = 12.214 Å, *b* = 3.0371 Å, *c* = 5.7981 Å, α = γ = 90° and β = 103.83° (Goldberg, 2001 ▸). The crystal studied in Fig. 5 ▸ was grown by the edge-defined film-fed growth method. During growth, unpredictable twinning can happen multiple times, even at very early stages in the seed region (Ueda *et al.*, 2016 ▸). As a result, the as-grown crystal may exhibit various orientations other than the intended one. In Fig. 5 ▸(*a*), the scattering of the Ga atoms is strong, producing strong background noise such that many of the weak diffraction spots are hardly visible.

Similarly to the above example, here we first select spots *A* and *C* to perform the LUT-P search, which gives 768 reflection pairs satisfying the interplanar angle 17.974° between *A* and *C*. Since β-Ga_2_O_3_ has many more allowed Bragg reflections than Si, here we set a smaller angular tolerance, Δθ = 0.1%. After saving the 768 pairs, we select spot *B* in Fig. 5 ▸(*a*) to perform LUT-P between *A* and *B*, which gives 996 reflection pairs. Next, the LUT-T filtering process is carried out between the two sets of reflection pairs, which leaves only ten pairs in the list of Fig. 5 ▸(*b*) that satisfy the reflection triplet conditions. Simulation with the first pair 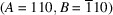 immediately gives the Laue pattern that matches the recorded image in Fig. 5 ▸(*b*). Meanwhile, the crystal orientation along the *z*′ axis (nearly perpendicular to the crystal surface) is determined as (0.0223, −1, −0.0903), which is 2.73° away from the 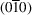 lattice plane. Here 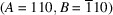 is the only solution, since trials of the other nine pairs do not produce matching patterns.

Obviously, LUT-T is a faster and more efficient procedure compared with the above G-rotation and LUT-P methods. Our numerous tests show that LUT-T is quite reliable and robust even if high-index spots are selected. To increase the success rate effectively while reducing the number of triplets in the list, the geometric parameters in Fig. 1 ▸, such as *D* and Φ, should be as accurate as possible (by means of calibration) so that the user can set a small angular tolerance and assign large values to *H*_max_, *K*_max_ and *L*_max_ to perform the search. However, inaccuracy in these parameters does not destabilize the process, and the user only needs to make the search over a broader range or try slightly different values for these parameters.

## 5. Summary

*LauePt* is a general-purpose application for simulating, indexing and analyzing Laue patterns. It has a single small executable file (∼2.5 MB) written with Visual C++ and Microsoft Foundation Classes. It can be run on any Windows computer or laptop without the need for large memory or powerful CPUs. Previous versions of *LauePt* do not have an efficient algorithm for recognizing or fitting Laue images taken from crystals of unknown orientation. In this paper we have introduced a major upgrade of *LauePt* to Version 4, to which three powerful search and fitting schemes have been added that completely overcome this difficulty.

The first scheme is a single-spot G-rotation method, which allows the user to select a single (low-index) diffraction spot from a Laue image and assign a trial reflection index **G** = *hkl* to it. *LauePt4* is then able to simulate all Laue patterns that have this reflection fixed along the specific direction in space. Determination of a 3D crystal orientation requires two directions. Here *LauePt4* allows the user to choose the other direction perpendicular to **G**, which corresponds to a 360° rotation of the crystal around **G**, to search for the correct simulation. The second scheme is a two-spot look-up method, LUT-P, that allows the user to select two diffraction spots. *LauePt4* then searches for all the reflection pairs in the user-defined range that satisfy the interplanar angle of these two spots. Afterwards, *LauePt4* can simulate the Laue patterns of all the candidate pairs to find the correct pair and simulation. The third scheme is a three-spot look-up method, LUT-T, in which *LauePt4* searches for reflection triplets satisfying the three interplanar angles between three user-selected diffraction spots and performs the simulation based on each triplet.

Our extensive tests show that the three schemes are all powerful for recognizing Laue patterns, with the LUT-T method being the most efficient. It can rapidly recognize almost any Laue images recorded from any crystal, even if the image quality is poor.

Overall, the current upgrade makes *LauePt4* a reliable crystallography tool with comprehensive functions. Since the schemes are implemented with highly efficient algorithms and optimized C++ code, the program size remains small and the running speed is extremely fast. Almost all search or simulation procedures (including intensity calculations) are executed and finished instantly without any noticeable delay. The fast speed and responsiveness of *LauePt4* greatly facilitate quick and arbitrary crystal rotation (with instant pattern update) and rapid screening of reflection pairs by simulations in LUT-P and LUT-T, even if the list contains thousands of pairs.

*LauePt4* is freely available for scientific use, and currently it can be downloaded from https://you.stonybrook.edu/xraycharacterization/lauept4/. It will also be uploaded to other public scientific software websites soon.

## Supplementary Material

Additional mathematical background. DOI: 10.1107/S1600576723007926/iu5045sup1.pdf
