# Supplementary material for: Upgraded LauePt4 for rapid recognition and fitting of Laue patterns from crystals with unknown orientations
Source: J Appl Crystallogr. 2023 Sep 27;56(Pt 5):1610–5. doi: 10.1107/S1600576723007926 (PMC10543676; doi:10.1107/S1600576723007926)
Supplement: Supplementary file 1 [file j-56-01610-sup1.pdf]

JOURNAL OF
APPLIED
CRYSTALLOGRAPHY

Volume 56 (2023)

Supporting information for article:

Upgraded *LauePt4* for rapid recognition and fitting of Laue patterns from crystals with unknown orientations

Vincent W. Huang, Yafei Liu, Balaji Raghothamachar and Michael Dudley

Crystal coordinate system

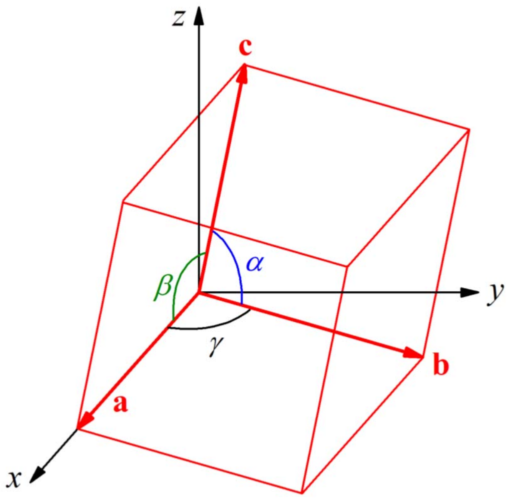

Fig. S1. Crystal coordinate system established on the unit cell of a crystal with lattice constants a, b, c, α, β, γ.

The *crystal* coordinate system (CS) *xyz* is defined in Fig. S1, where the *x*-axis is parallel to **a**, the *y*-axis is in the **ab** plane, and the *z*-axis is perpendicular to the **ab** plane. Then the three primitive lattice vectors can be expressed in the *xyz* CS as

$$\mathbf{a} = a_x\hat{\mathbf{x}} = a\hat{\mathbf{x}}, \quad (1a)$$

$$\mathbf{b} = b_x\hat{\mathbf{x}} + b_y\hat{\mathbf{y}} = b\cos\gamma\,\hat{\mathbf{x}} + b\sin\gamma\,\hat{\mathbf{y}}, \quad (1b)$$

$$\mathbf{c} = c_x\hat{\mathbf{x}} + c_y\hat{\mathbf{y}} + c_z\hat{\mathbf{y}}, \quad (1c)$$

with $c_x = c\cos\beta$, $c_y = c\,(\cos\alpha - \cos\beta\cos\gamma)/\sin\gamma$, and $c_z = \sqrt{c^2 - c_x^2 - c_y^2}$. Here $\hat{\mathbf{x}}$, $\hat{\mathbf{y}}$, $\hat{\mathbf{z}}$ are unit vectors along the *x*, *y*, *z* axes of the crystal CS, respectively. Then the reciprocal lattice vectors are

$$\mathbf{a}^* = a_x^*\hat{\mathbf{x}} + a_y^*\hat{\mathbf{y}} + a_z^*\hat{\mathbf{z}} = \frac{b_y c_z}{V}\hat{\mathbf{x}} - \frac{b_x c_z}{V}\hat{\mathbf{y}} + \frac{b_x c_y - b_y c_x}{V}\hat{\mathbf{z}}, \quad (2a)$$

$$\mathbf{b}^* = b_y^*\hat{\mathbf{y}} + b_z^*\hat{\mathbf{z}} = \frac{a_x c_z}{V}\hat{\mathbf{y}} - \frac{a_x c_y}{V}\hat{\mathbf{z}}, \quad (2b)$$

$$\mathbf{c}^* = c_z^*\hat{\mathbf{z}} = \frac{a_x b_y}{V}\hat{\mathbf{z}}, \quad (2c)$$

where $V = abc_z\sin\gamma$ is the volume of the unit cell.

Expressing the primitive reciprocal lattice vectors a*, b*, c* in the laboratory coordinate systems

As shown in Fig. 1 of the paper, when a user clicks on a spot $P\ (x_p, y_p)$ of the Laue image on the CCD plane, the diffracted wavevector of this spot is along the vector

$$\mathbf{k}_g = -x_p\hat{\mathbf{x}}' + r_p\sin(\Phi+\varphi)\,\hat{\mathbf{y}}' + r_p\cos(\Phi+\varphi)\,\hat{\mathbf{z}}' \tag{3}$$

in the *laboratory coordinate system* $x'y'z'$, where $\hat{\mathbf{x}}'$, $\hat{\mathbf{y}}'$, $\hat{\mathbf{z}}'$ are unit vectors along the x', y', z' axes, respectively. Here only the direction is valid, and we calculate the unit vector to represent the direction, $\hat{\mathbf{k}}_g = \mathbf{k}_g/|\mathbf{k}_g|$. The unit vector along the incidence direction is $\hat{\mathbf{k}}_0 = -\hat{\mathbf{z}}'$. The diffraction vector **G** in Fig. 1(b) of the paper is parallel to the vector $\hat{\mathbf{k}}_g - \hat{\mathbf{k}}_0$. We can obtain the unit vector

$$\hat{\mathbf{G}}' = (\hat{\mathbf{k}}_g - \hat{\mathbf{k}}_0)/|\hat{\mathbf{k}}_g - \hat{\mathbf{k}}_0| = \hat{G}_{x'}\hat{\mathbf{x}}' + \hat{G}_{y'}\hat{\mathbf{y}}' + \hat{G}_{z'}\hat{\mathbf{z}}' \tag{4}$$

along the direction of **G** in the $x'y'z'$ laboratory coordinate system. Meanwhile, we need to set another two axes $\hat{\mathbf{U}}'$ and $\hat{\mathbf{V}}'$ such that $\hat{\mathbf{U}}'$, $\hat{\mathbf{V}}'$, and $\hat{\mathbf{G}}'$ form an intermediate right-handed orthogonal coordinate system (see Fig. S2). Here we choose

$$\hat{\mathbf{U}}' = \frac{\hat{\mathbf{z}}' \times \hat{\mathbf{G}}'}{|\hat{\mathbf{z}}' \times \hat{\mathbf{G}}'|} = \hat{U}_{x'}\hat{\mathbf{x}}' + \hat{U}_{y'}\hat{\mathbf{y}}' + \hat{U}_{z'}\hat{\mathbf{z}}' \tag{5a}$$

and

$$\hat{\mathbf{V}}' = \hat{\mathbf{G}}' \times \hat{\mathbf{U}}' = \hat{V}_{x'}\hat{\mathbf{x}}' + \hat{V}_{y'}\hat{\mathbf{y}}' + \hat{V}_{z'}\hat{\mathbf{z}}'. \tag{5b}$$

Note that for exact back reflection, $\hat{\mathbf{G}}' = \hat{\mathbf{z}}'$. For this special case, we define $\hat{\mathbf{U}}' = \hat{\mathbf{x}}'$ and $\hat{\mathbf{V}}' = \hat{\mathbf{y}}'$.

Now in the **G**-rotation method, if the user assigns an index *hkl* to **G**, in the *crystal coordinate system* we have

$$\mathbf{G} = h\mathbf{a}^* + k\mathbf{b}^* + l\mathbf{c}^* \tag{6}$$

and the unit vector along **G** is $\hat{\mathbf{G}} = \mathbf{G}/|\mathbf{G}|$. Afterwards, *LauePt4* will automatically choose a vector

$$\mathbf{G}_u = h_u\mathbf{a}^* + k_u\mathbf{b}^* + l_u\mathbf{c}^* \tag{7}$$

perpendicular to **G**. Note that to ensure $\mathbf{G}_u$ is perpendicular to **G**, here h_u, k_u, l_u may not necessarily be integers (but h, k , l are always integers). Then *LauePt4* calculates a third vector $\mathbf{G}_v = \mathbf{G} \times \mathbf{G}_u$ that is perpendicular to both **G** and $\mathbf{G}_u$. The three vectors are then normalized to three unit vectors

$$\hat{\mathbf{G}}_u = (\hat{\mathrm{G}}_{ux}\ \ \hat{\mathrm{G}}_{uy}\ \ \hat{\mathrm{G}}_{uz}), \tag{8a}$$

$$\hat{\mathbf{G}}_v = (\hat{\mathrm{G}}_{vx}\ \ \hat{\mathrm{G}}_{vy}\ \ \hat{\mathrm{G}}_{vz}), \tag{8b}$$

$$\hat{\mathbf{G}} = (\hat{\mathrm{G}}_{x}\ \ \hat{\mathrm{G}}_{y}\ \ \hat{\mathrm{G}}_{z}) \tag{8c}$$

in the *crystal* coordinate system. The purpose of this $\hat{\mathbf{G}}_u\hat{\mathbf{G}}_v\hat{\mathbf{G}}$ system is that we want to express the three reciprocal lattice vectors $\mathbf{a}^*$, $\mathbf{b}^*$, $\mathbf{c}^*$ in this system:

$$\mathbf{a}^* = a_u^*\hat{\mathbf{G}}_u + a_v^*\hat{\mathbf{G}}_v + a_G^*\hat{\mathbf{G}}, \quad (9a)$$

$$\mathbf{b}^* = b_u^*\hat{\mathbf{G}}_u + b_v^*\hat{\mathbf{G}}_v + b_G^*\hat{\mathbf{G}}, \quad (9b)$$

$$\mathbf{c}^* = c_u^*\hat{\mathbf{G}}_u + c_v^*\hat{\mathbf{G}}_v + c_G^*\hat{\mathbf{G}}\mathrm{a} \quad (9c)$$

with

$$a_u^* = \mathbf{a}^*\cdot\hat{\mathbf{G}}_u \quad a_v^* = \mathbf{a}^*\cdot\hat{\mathbf{G}}_v \quad a_G^* = \mathbf{a}^*\cdot\hat{\mathbf{G}}, \quad (10a)$$

$$b_u^* = \mathbf{b}^*\cdot\hat{\mathbf{G}}_u \quad b_v^* = \mathbf{b}^*\cdot\hat{\mathbf{G}}_v \quad b_G^* = \mathbf{b}^*\cdot\hat{\mathbf{G}} \quad (10b)$$

$$c_u^* = \mathbf{c}^*\cdot\hat{\mathbf{G}}_u \quad c_v^* = \mathbf{c}^*\cdot\hat{\mathbf{G}}_v \quad c_G^* = \mathbf{c}^*\cdot\hat{\mathbf{G}} \quad (10c)$$

All the coefficients in Eqs. (10a, 10b, 10c) can be calculated using Eqs. (2a, 2b, 2c, 8a, 8b, 8c) in the *crystal* coordinate system.

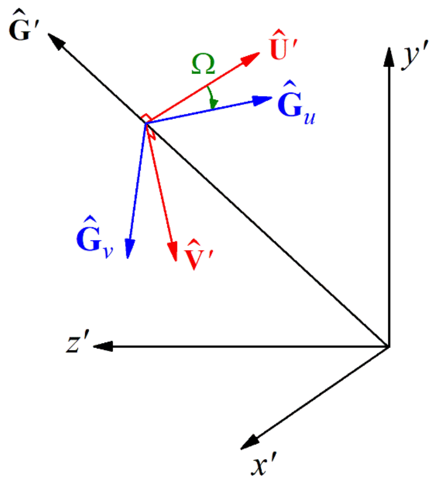

Fig. S2. The intermediate coordinate system shown in the $x'y'z'$ laboratory coordinate system. $\hat{\mathbf{U}}'$ $\hat{\mathbf{V}}'$ $\hat{\mathbf{G}}'$ are orthogonal unit vectors form the right-handed intermediate coordinate system. $\hat{\mathbf{G}}_u$ $\hat{\mathbf{G}}_v$ $\hat{\mathbf{G}}'$ also form a right-handed orthogonal coordinate system, but the $\hat{\mathbf{G}}_u$ $\hat{\mathbf{G}}_v$ $\hat{\mathbf{G}}'$ system is rotated from $\hat{\mathbf{U}}'$ $\hat{\mathbf{V}}'$ $\hat{\mathbf{G}}'$ around $\hat{\mathbf{G}}'$ by an angle Ω.

Next we move to the *laboratory coordinate system*. In this system, $\hat{\mathbf{G}}$ is aligned to be the same as $\hat{\mathbf{G}}'$ in Eq. (4). $\hat{\mathbf{G}}_u$ and $\hat{\mathbf{G}}_v$ are both set to be in the plane defined by $\hat{\mathbf{U}}'$ and $\hat{\mathbf{V}}'$, but are rotated around $\hat{\mathbf{G}}$ by an angle Ω, see Fig. S2. Here the $\hat{\mathbf{G}}$ vector is fixed in space. This corresponds to rotation of the crystal along the $\hat{\mathbf{G}}$ vector. Then $\hat{\mathbf{G}}_u$ $\hat{\mathbf{G}}_v$ $\hat{\mathbf{G}}$ can be expressed as

$$\hat{\mathbf{G}}_u = \cos\Omega\hat{\mathbf{U}}' + \sin\Omega\,\hat{\mathbf{V}}'. \quad (11a)$$

$$\hat{\mathbf{G}}_v = -\sin\Omega\hat{\mathbf{U}}' + \cos\Omega\,\hat{\mathbf{V}}'. \quad (11b)$$

$$\hat{\mathbf{G}} = \hat{\mathbf{G}}' \tag{11c}$$

Now if we replace $\hat{\mathbf{G}}_u$ $\hat{\mathbf{G}}_v$ $\hat{\mathbf{G}}$ in Eqs. (11a, 11b, 11c) with those in Eq. (4, 5a, 5b) and then insert Eqs. (11a, 11b, 11c) into Eqs. (9a, 9b, 9c), we obtain the expressions of $\mathbf{a}^*$, $\mathbf{b}^*$, $\mathbf{c}^*$ in the laboratory coordinate system as

$$\mathbf{a}^* = a^*_{x'}\hat{\mathbf{x}}' + a^*_{y'}\hat{\mathbf{y}}' + a^*_{z'}\hat{\mathbf{z}}'\mathrm{a} \tag{12a}$$

$$\mathbf{b}^* = b^*_{x'}\hat{\mathbf{x}}' + b^*_{y'}\hat{\mathbf{y}}' + b^*_{z'}\hat{\mathbf{z}}' \tag{12b}$$

$$\mathbf{c}^* = c^*_{x'}\hat{\mathbf{x}}' + c^*_{y'}\hat{\mathbf{y}}' + c^*_{z'}\hat{\mathbf{z}}' \tag{12c}$$

The nine coefficients, $a^*_{x'}$ $a^*_{y'}$ $a^*_{z'}$ $b^*_{x'}$ $b^*_{y'}$ $b^*_{z'}$ $c^*_{x'}$ $c^*_{y'}$ $c^*_{z'}$ are all the geometrical parameters *LauePt4* requires for calculating any diffraction vectors in the laboratory coordinate system during simulation. Based on the six lattice parameters, these coefficients only depend on the *hkl* indices in Eq. (6) and the rotation angle Ω in Eqs. (11a) and (11b). The nine coefficients also depend on the automatically chosen indices h_u, k_u, l_u in Eq. (7), but h_u, k_u, l_u only determine the starting orientation $\Omega = 0$ of the crystal rotation. Rotation of Ω from 0 to 360° covers all the possible $\hat{\mathbf{G}}_u$ vectors perpendicular to the fixed $\mathbf{G}$ no matter what *LauePt4* automatically chooses for h_u, k_u, l_u.

In the LUT-P method, after the user selects two diffraction spots in the Laue image, *LauePt4* calculates the unit vectors $\hat{\mathbf{H}}'_1$ and $\hat{\mathbf{H}}'_2$ along the two corresponding diffraction vectors using Eqs. (3) and (4) in the *laboratory coordinate system*. After the user selects a reflection pair ($h_1\ k_1\ l_1,\ h_2\ k_2\ l_2$) from the matching list, the two diffraction vectors in the *crystal coordinate system* are also determined:

$$\mathbf{H}_1 = h_1\mathbf{a}^* + k_1\mathbf{b}^* + l_1\mathbf{c}^* \tag{13a}$$

$$\mathbf{H}_2 = h_2\mathbf{a}^* + k_2\mathbf{b}^* + l_2\mathbf{c}^* \tag{13b}$$

LauePt4 then makes these five changes: (i) Replace $\hat{\mathbf{G}}'$ in Eq. (4) with the current $\hat{\mathbf{H}}'_1$. (ii) Replace $\hat{\mathbf{U}}'$ in Eq. (5a) with

$$\hat{\mathbf{U}}' = \frac{\hat{\mathbf{H}}'_1 \times \hat{\mathbf{H}}'_2}{|\hat{\mathbf{H}}'_1 \times \hat{\mathbf{H}}'_2|} = \hat{U}_{x'}\hat{\mathbf{x}}' + \hat{U}_{y'}\hat{\mathbf{y}}' + \hat{U}_{z'}\hat{\mathbf{z}}' \tag{14}$$

(iii) Replace $\mathbf{G}$ in Eq. (6) with $\mathbf{H}_1$ in Eq. (13a). (iv) Replace $\mathbf{G}_u$ in Eq. (7) with the vector $\mathbf{H}_1 \times \mathbf{H}_2$ calculated by using Eqs. (13a) and (13b), and (v) set $\Omega = 0$ in Eqs. (11a) and (11b) (because no crystal rotation is allowed in the two-spot LUT-P method). After these changes, *LauePt4* repeats all the above calculations for $a^*_{x'}$ $a^*_{y'}$ $a^*_{z'}$ $b^*_{x'}$ $b^*_{y'}$ $b^*_{z'}$ $c^*_{x'}$ $c^*_{y'}$ $c^*_{z'}$ and then simulates the specific Laue pattern.

Overall, when the user clicks to select a spot on the screen (in the Laue image), the direction of corresponding diffraction vector $\mathbf{G}$ is determined in the laboratory coordinate system. When the reflection is assigned a reflection index, the expression of $\mathbf{G}$ in the crystal coordinate system is determined. But the

two expressions refer to the same vector in space. This identity bridges the crystal coordinate system and the laboratory coordinate system. But full bridging requires two directions. In the LUT-P method, the **G** vector is $\mathbf{G}_1$, and the second direction is the vector $\mathbf{G}_1 \times \mathbf{G}_2$ that is always perpendicular to $\mathbf{G}_1$. In the G-rotation method, the second direction in the laboratory coordinate system is $\hat{\mathbf{z}}' \times \widehat{\mathbf{G}}'$, and in the crystal coordinate system, *LauePt4* automatically selects a direction $(h_u\ k_u\ l_u)$ but leaves the freedom to rotate this direction around **G** by 360°.
